# A Regulatory Circuit Composed of a Transcription Factor, IscR, and a Regulatory RNA, RyhB, Controls Fe-S Cluster Delivery

**DOI:** 10.1128/mBio.00966-16

**Published:** 2016-09-20

**Authors:** Pierre Mandin, Sylvia Chareyre, Frédéric Barras

**Affiliations:** CNRS-LCB, UMR7283, Aix-Marseille Université, Marseille, France

## Abstract

Fe-S clusters are cofactors conserved through all domains of life. Once assembled by dedicated ISC and/or SUF scaffolds, Fe-S clusters are conveyed to their apo-targets via A-type carrier proteins (ATCs). *Escherichia coli* possesses four such ATCs. ErpA is the only ATC essential under aerobiosis. Recent studies reported a possible regulation of the *erpA* mRNA by the small RNA (sRNA) RyhB, which controls the expression of many genes under iron starvation. Surprisingly, *erpA* has not been identified in recent transcriptomic analysis of the iron starvation response, thus bringing into question the actual physiological significance of the putative regulation of *erpA* by RyhB. Using an sRNA library, we show that among 26 sRNAs, only RyhB represses the expression of an *erpA-lacZ* translational fusion. We further demonstrate that this repression occurs during iron starvation. Using mutational analysis, we show that RyhB base pairs to the *erpA* mRNA, inducing its disappearance. In addition, IscR, the master regulator of Fe-S homeostasis, represses expression of *erpA* at the transcriptional level when iron is abundant, but depleting iron from the medium alleviates this repression. The conjunction of transcriptional derepression by IscR and posttranscriptional repression by RyhB under Fe-limiting conditions is best described as an incoherent regulatory circuit. This double regulation allows full expression of *erpA* at iron concentrations for which Fe-S biogenesis switches from the ISC to the SUF system. We further provide evidence that this regulatory circuit coordinates ATC usage to iron availability.

## IMPORTANCE

Regulatory small RNAs (sRNAs) have emerged as major actors in the control of gene expression in the last few decades. Relatively little is known about how these regulators interact with classical transcription factors to coordinate genetic responses. We show here how an sRNA, RyhB, and a transcription factor, IscR, regulate expression of an essential gene, *erpA*, in the bacterium *E. coli*. ErpA is involved in the biogenesis of Fe-S clusters, an important class of cofactors involved in a plethora of cellular reactions. Interestingly, we show that RyhB and IscR repress expression of *erpA* under opposite conditions in regard to iron concentration, forming a regulatory circuit called an “incoherent network.” This incoherent network serves to maximize expression of *erpA* at iron concentrations where it is most needed. Altogether, our study paves the way for a better understanding of mixed regulatory networks composed of RNAs and transcription factors.

## INTRODUCTION

Iron-sulfur (Fe-S) clusters containing proteins control a wide variety of biological processes, including metabolism, respiration, photosynthesis, DNA repair, and gene regulation (1–3). Two multiprotein complexes, ISC and SUF, catalyze *in vivo* assembly and delivery of Fe-S clusters in both prokaryotes and eukaryotes (4, 5). The model organism *Escherichia coli* contains both of these systems. In this bacterium, the current prevailing hypothesis is that the ISC system serves as a housekeeping system, while the SUF system functions under stress-inducing conditions, such as oxidative stress and iron scarcity (6, 7).

Although the two systems rely on different proteins, they build and deliver clusters using related molecular mechanisms (reviewed in references 4, 5, and 8). A cysteine desulfurase provides sulfur from the free cysteine cellular pool, while a dedicated specific iron source, if any, remains to be identified. The Fe-S cluster is transiently assembled onto a scaffold complex and is then transferred to A-type carriers (ATCs) that deliver the Fe-S clusters to apoproteins. Four of these ATC proteins have been described in *E. coli*: IscA and SufA, encoded by the *isc* and *suf* operons, respectively, and NfuA and ErpA, which are encoded elsewhere on the chromosome (9, 10).

In *E. coli*, the ATC proteins are biochemically redundant (11). The current model is that ATC diversity provides different transfer routes from the assembly machineries to the apoproteins as a function of environmental conditions and, possibly, targets to be matured. Remarkably, ErpA is the only essential ATC when *E. coli* grows under aerobic conditions because it transfers an Fe-S cluster to the IspG/H proteins that catalyze the synthesis of the essential isoprenoid precursors (10). However, the requirement of ErpA for maturation of IspG/H can be bypassed when the demand for Fe-S clusters is lowered. Thus, under anaerobiosis, IscA can also mediate Fe-S transfer to IspG/H, making both ErpA and IscA functionally redundant under such conditions. Meanwhile, under iron limitation SufA was proposed to be the only ATC able to ensure Fe-S cluster transfer to IspG/H (11). Hence, starting from a set of biochemically related ATCs, the cell appears to use genetic regulation likely to exploit functional ATC specialization that has not been revealed by biochemical studies. In particular, one might surmise that under iron limitation, it would be counterproductive to produce functionally redundant iron-using ATCs.

In *E. coli*, the major regulator of Fe-S homeostasis is the IscR transcription factor, which itself contains a [2Fe-2S] cluster (12–14). IscR is encoded by the first gene of the *iscRSUA* operon. Both apo and holo forms of IscR are active as transcriptional regulators; however, the IscR cluster ligation state modifies the DNA recognition properties (15, 16). Holo-IscR binds and represses expression at so-called type 1 promoters, which include the *isc* operon and the *erpA* and the *nfuA* promoters. Type 2 promoters can be bound by both apo- and holo-IscR. In particular, the *suf* promoter is a type 2 promoter at which IscR binds and activates transcription (7). Thus, IscR represses expression of the *iscRSUA* operon when Fe-S demand is fulfilled, while it activates expression of the *suf* operon under conditions that are detrimental for Fe-S formation, such as oxidative stress or iron scarcity.

The regulators Fur and RyhB also play an important role in controlling Fe-S biogenesis (17). Fur, the iron sensor regulator, represses transcription of several genes involved in iron import and metabolism when it is bound to Fe (18). Fur also represses expression of the noncoding RNA (small RNA [sRNA]) RyhB. Under iron limitation, Fur repression is alleviated, and RyhB is synthesized and regulates, mostly negatively, the expression of more than 50 genes, the majority of which encode iron-containing proteins (19). Thus, RyhB is thought to reallocate bioavailable iron to essential targets, helping the cell to cope with iron scarcity (20). RyhB base pairs to the *iscRSUA* mRNA upstream of the *iscS* gene, inducing the degradation of the 3′ part of the mRNA, while the 5′ part containing *iscR* remains stable (17). In this way, RyhB favors the use of the Suf system during iron starvation. Notably, Fur also represses the *suf* operon, encoding the Suf system, thus contributing to switching from ISC to SUF under those conditions (21).

RyhB was recently predicted to base pair to the *erpA* mRNA, and the *erpA* mRNA was copurified with MS2-tagged RyhB (22, 23). Surprisingly, while regulation of *erpA* by RyhB seemed very likely, global transcriptomic and deep sequencing approaches have failed to identify *erpA* as a target of RyhB (20, 24). Therefore, the question of the physiological regulation of *erpA* by RyhB remained to be addressed. We first here definitely established that RyhB indeed regulates *erpA* expression by base pairing to its mRNA and inducing its disappearance. We further show that IscR represses expression of *erpA* under opposite conditions than those of RyhB and that alleviation of repression by IscR masks the effect of RyhB under severely iron-limited conditions. Thus, these regulators form an incoherent circuit that controls *erpA* expression as a function of iron availability. The net outcome is a bimodal behavior, which culminates in the induction of *erpA* expression in a narrow range of iron concentrations. While establishing a case of a mixed circuit, including both an sRNA and a transcriptional regulator, this study provides a framework to describe how *E. coli* exploits the biochemical properties of ATCs while preventing redundancy to ensure Fe-S cluster delivery throughout a broad range of iron concentrations.

## RESULTS

### RyhB represses *erpA* expression.

**FIG 1:**
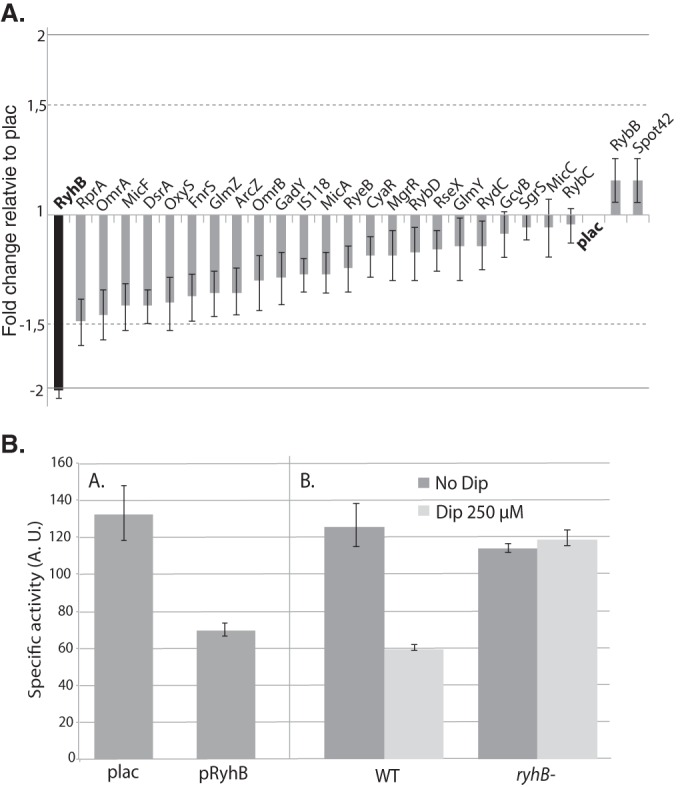
RyhB represses expression of *erpA*. (A) A plasmid library allowing the expression of 26 sRNAs, as well as the empty vector, was transformed in the strain containing the P_BAD_-*erpA-lacZ* fusion. The effect of the overexpression of each sRNA expressed individually was measured by growing the cells for 6 h in LB containing 100 µg/ml ampicillin, 0.02% arabinose, and 100 µM IPTG, after which β-galactosidase activity was measured. Results are represented as a function of the fold change in activity of the fusion for each overexpressing plasmid compared to the plac empty vector. Dashed lines represent the threshold that was chosen to consider significant effects. Error bars represent standard deviations for a total of 8 independent experiments. (B) (Left) The P_BAD_-*erpA*-*lacZ*-containing strain was transformed with the empty plac vector or with the pRyhB plasmid containing *ryhB* under the control of an IPTG-inducible promoter. Strains were grown in flasks containing LB with 100 µg/ml ampicillin, 0.02% arabinose, and 100 µM IPTG for 6 h, after which β-galactosidase activity was measured. (Right) The strain containing the P_BAD_-*erpA*-*lacZ* fusion and a *ryhB* isogenic mutant were cultivated in LB with or without 250 µM DIP for 6 h before β-galactosidase activity was measured. Arbitrary units were empirically determined to be approximately equivalent to Miller units. Each point represents the mean from 8 or more experiments; error bars represent the standard deviations.

We first wanted to test if expression of *erpA* was regulated by RyhB and/or other sRNAs. To do so, we used a library of plasmids, containing 26 different *E. coli* sRNAs under the control of a P_lac_ promoter, to test the effect of overexpressing these sRNAs on a P_BAD_-*erpA-lacZ* fusion (Fig. 1A) (25, 26). This fusion was placed under an inducible promoter to avoid the indirect effect of overexpressing the sRNAs on transcription of the fusion. Only the overexpression of RyhB had a significant effect by inducing a 2-fold repression of the activity compared to the empty vector (Fig. 1A). The same 2-fold-repressing effect of RyhB on the fusion could also be observed when cells were grown in culture flasks (Fig. 1B, left). In agreement with previous studies, these data indicated that RyhB was the only sRNA involved in *erpA* regulation.

Under physiological conditions, RyhB is normally expressed under iron-limiting conditions due to loss of Fur regulation (19). As regulation of *erpA* expression has been tested only through overexpression of the sRNA (22), we tested if iron starvation repressed activity of the P_BAD_-*erpA-lacZ* fusion in a RyhB-dependent manner. To do so, wild-type (WT) or *ryhB* mutant cells containing the fusion were grown in the presence or absence of 250 µM 2,2′-dipyridyl (DIP), a routinely used iron chelator, before measurement of β-galactosidase activity (Fig. 1B, right). No significant difference could be seen between the WT and the *ryhB* mutant in the absence of iron chelation. In contrast, the activity of the fusion was diminished by 2-fold when the WT strain was grown in the presence of 250 µM DIP but was unaltered in the *ryhB* mutant (Fig. 1B). We thus concluded that RyhB represses the activity of P_BAD_-*erpA-lacZ* during iron starvation*.*

### RyhB base pairs to the *erpA* mRNA.

**FIG 2:**
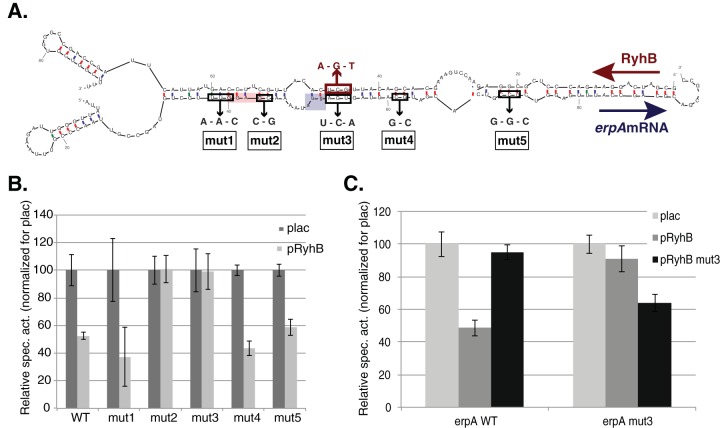
RyhB base pairs to the *erpA* mRNA. (A) Base pairing predicted between RyhB and the *erpA* mRNA. Nucleotides belonging to RyhB are represented on top, and the ones corresponding to the *erpA* mRNA sequence are represented on the bottom; positions relative to the RyhB or *erpA* transcriptional start site are indicated above or below the sequences, respectively. The putative ribosome binding site and the AUG start codon of *erpA* are represented by the pink and blue boxes, respectively. Mutations introduced in the *erpA* mRNA used in panel B are denoted with arrows. (B) Cells containing either the WT P_BAD_-*erpA-lacZ* fusion or the mutated versions of the fusion were transformed with the empty plac vector (dark gray bars) or the pRyhB plasmid (light gray bars) and grown in LB containing 100 µg/ml ampicillin, 0.02% arabinose, and 100 µM IPTG for 6 h, after which β-galactosidase activity was measured. (C) Cells containing either the WT P_BAD_-*erpA-lacZ* fusion (left set of bars) or the mut3 mutated version of the fusion (right set of bars) were transformed with the empty plac vector (light gray bars), the pRyhB plasmid (dark gray bars), or the pRyhB-mut3 mutant (black bars), and β-galactosidase activity was measured as described for panel B. Each point represents the mean from 8 or more experiments; error bars represent standard deviations.

If RyhB’s regulation of *erpA* expression is direct, base pairing between both *erpA* and RyhB transcripts is expected. We predicted a large base pairing involving almost all of the first 65 nucleotides (nt) of RyhB and 57 nt of the *erpA* mRNA using the Mfold program (Fig. 2A). Interestingly, this base-pairing region encompasses the ribosome binding site (RBS) and the AUG codon of *erpA*. A similar prediction had been proposed by Wright et al. using the target prediction program CopraRNA (22).

We introduced a series of five mutations in the *erpA*-*lacZ* fusion, named mut1 to mut5, predicted to disrupt the base pairing depicted above (Fig. 2A). Overexpression of RyhB failed to repress activity of *erpA*_mut2_ and *erpA*_mut3_ alleles but repressed expression of the 3 other fusion variants (Fig. 2B). This result indicated that while extensive base pairing can be predicted between the two RNAs, only the region of base pairing close to the translation initiation region seems required for effective regulation.

Next, we introduced a mutation in the plasmid carrying the *ryhB* gene such that it would disrupt base pairing with the WT *erpA* mRNA but restore complementarity to the *erpA*_mut3_ allele. The mutated allele of RyhB repressed (1.5-fold) the *erpA*_mut3_-*lacZ* fusion, while it did not repress the activity of the WT *erpA-lacZ* fusion (Fig. 2C). We concluded that RyhB represses expression of *erpA* directly by RNA/RNA base pairing close to the *erpA* translation initiation region.

### Overexpression of RyhB induces the disappearance of the *erp*A mRNA.

**FIG 3:**
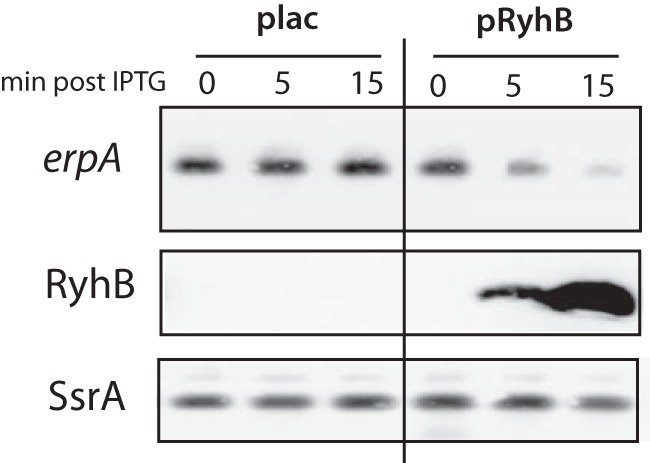
RyhB induces the disappearance of the *erpA* mRNA. The MG1655 *ΔryhB* strain was transformed with the empty plac vector or with the pRyhB plasmid containing *ryhB* under the control of an IPTG-inducible promoter. Strains were grown in LB, and at an OD_600_ of 0.5, samples were taken and 100 µM IPTG was immediately added to the culture (time zero). Samples were then taken at 5, 10, and 15 min. Total RNA was extracted and submitted to a Northern blotting experiment with probes directed against the *erpA* mRNA, RyhB, or SsrA (bottom panels).

In many cases, RyhB induces the degradation of its mRNA targets through the recruitment of RNase E (27). We thus performed Northern blotting experiments to test the effect of RyhB overexpression on the *erpA* mRNA. The pRyhB plasmid or the empty vector was transformed into a *ryhB*-deletion strain. Strains were grown to an optical density at 600 nm (OD_600_) of 0.5, at which point synthesis of RyhB was induced with 100 µM isopropyl-β-d-thiogalactopyranoside (IPTG). The production of RyhB induced a rapid disappearance of the *erpA* mRNA (Fig. 3), while it remained stable in the strain transformed with the empty vector. Altogether, these data allowed us to conclude that RyhB binding induces a disappearance of the *erpA* mRNA, most likely by destabilization.

### RyhB and IscR cancel each other’s effect during severe iron starvation.

In our previous experiments, expression of the *erpA-lacZ* fusion was driven by an inducible P_BAD_ promoter. As the *erpA* promoter has previously been shown to be transcriptionally repressed by holo-IscR (15), we wanted to study *erpA* expression when both IscR and RyhB regulation can act concomitantly. Hence, we designed a new *erpA-lacZ* fusion, which contained the RyhB binding region and the endogenous *erpA* promoter, including the previously described IscR binding site. The resulting P*_erpA_*-*lacZ*-containing strain was grown in LB with or without 250 µM DIP.

**FIG 4:**
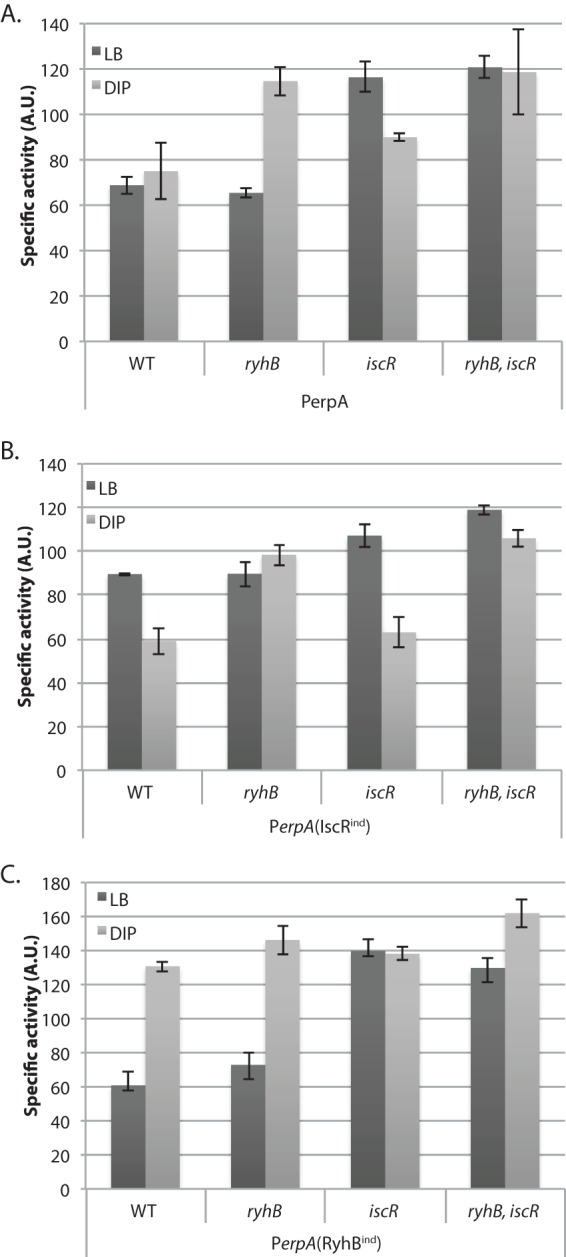
RyhB and IscR regulate *erpA* expression during iron starvation. Strains containing either the P*_erpA_-lacZ* (A), the P_erpA_(IscR^ind^) (B), or the P_erpA_(RyhB^ind^) (C) fusion and their *ryhB*, *iscR*, or double *ryhB iscR* isogenic mutants were assayed for β-galactosidase activity. Strains were grown for 6 h in LB medium with (light gray bars) or without (dark gray bars) 250 µM DIP before activity was measured. Each point represents the mean from 8 or more experiments; error bars represent the 95% confidence intervals of the means.

In striking contrast to the P_BAD_-driven fusion that was repressed by RyhB when cells were treated with 250 µM DIP, the same treatment did not result in any significant change in activity of the P*_erpA_-lacZ* fusion (Fig. 4A). However, deleting *ryhB* increased P*_erpA_-lacZ* expression upon DIP treatment, confirming that RyhB repressed expression of *erpA* during iron starvation. Furthermore, deleting *iscR* from the chromosome increased the activity of the P*_erpA_-lacZ* fusion-containing strain grown under iron-replete conditions (Fig. 4A), confirming repression of *erpA* expression by IscR. As expected, deleting both *iscR* and *ryhB* resulted in maximal expression of the fusion in the presence or absence of DIP. We thus hypothesized that, in the presence of DIP, IscR alleviation of repression at P_erpA_ was canceled by posttranscriptional repression by RyhB.

To test this hypothesis, we constructed two new mutated versions of the P*_erpA_-lacZ* fusion by introducing *cis*-acting mutations in its control region. The first allele had an altered IscR binding site following modification of nucleotides −19 to −21 (GGG to CCC) (16). Activity of this fusion, named P_erpA_(IscR^ind^) (for IscR independent), was only modestly augmented upon deletion of *iscR* (Fig. 4B), confirming that the mutation had severely affected the ability of IscR to repress the P*_erpA_-lacZ* fusion promoter. Expression of the P_erpA_(IscR^ind^)-*lacZ* fusion was decreased by 2-fold in WT or *iscR* mutant cells grown in the presence of 250 µM DIP. However, this effect was completely abolished when a *ryhB* mutation was introduced in both of these strains. Thus, a *cis* mutation preventing IscR repression clearly revealed RyhB-mediated regulation of *erpA* under iron-limiting growth conditions.

The second allele had its RyhB binding site containing the mut3 mutation described above and was named P_erpA_(RyhB^ind^) (for RyhB independent) (Fig. 2A). Activity of this fusion was enhanced 2-fold upon addition of 250 µM DIP both in the wild type and in the *ryhB* mutant (Fig. 4C). This indicated that P_erpA_(RyhB^ind^) was not regulated by RyhB. However, expression of this fusion was still regulated by IscR, as deleting *iscR* yielded a 2-fold increase whether DIP was added or not.

Altogether, these results showed that under severely iron-limited conditions, RyhB-mediated repression is compensated by alleviation of IscR repression.

### Coregulation by RyhB and IscR permits expression of *erpA* in a defined range of iron concentrations.

**FIG 5:**
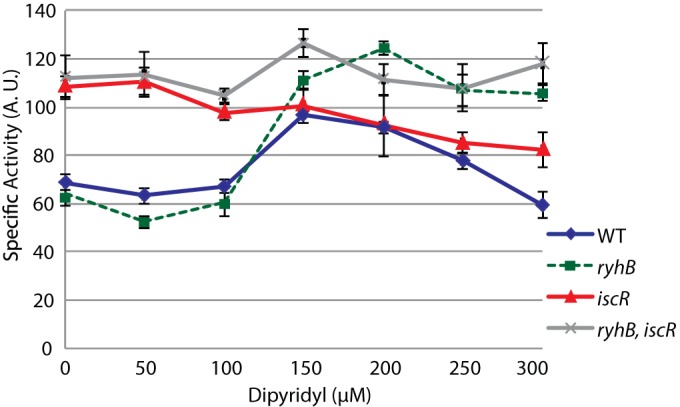
RyhB and IscR allow full expression of *erpA* in a defined iron concentration range. The strains containing the P*_erpA_-lacZ* fusion and its *ryhB*, *iscR*, and *ryhB iscR* isogenic derivative mutants were grown for 6 h in various concentrations of DIP before cells were lysed and β-galactosidase activity was measured. Results are expressed in arbitrary units that were empirically determined to be approximately equivalent to Miller units; each point represents the mean from 8 or more experiments; error bars represent the 95% confidence intervals of the means.

The dual regulation by RyhB and IscR of *erpA* reminded us of an incoherent circuit in which a common signal gives rise to two antagonistic effects on one target gene (28). Such circuits have been shown to provoke bimodal expression of genes that peak at certain concentrations of inducer (29). We thus aimed at identifying if there was a given concentration of iron that would favor maximal expression of *erpA* by canceling both IscR and RyhB repression. To this end, cells containing the wild-type P*_erpA_*-*lacZ* fusion were grown in LB supplemented with increasing concentrations of DIP, from 0 to 300 µM. The *ryhB*, *iscR*, and *ryhB iscR* isogenic mutants were likewise assayed, and the results are presented in Fig. 5.

In the WT strain, P*_erpA_*-*lacZ* expression increased with DIP concentration from 0 to 150 µM, after which expression of the fusion went down. At 300 µM DIP, the activity of the fusion was similar to that of untreated cells (Fig. 5). The *ryhB* mutant profile was identical to that of the WT strain for concentrations of DIP of ≤150 µM. In contrast to the WT strain, though, in the *ryhB* mutant, expression of the P*_erpA_*-*lacZ* fusion remained high at elevated DIP concentrations (Fig. 5).

In the *iscR* mutant, P*_erpA_*-*lacZ* expression was maximal in the absence of DIP and decreased above 100 µM DIP to reach minimal WT-like levels (Fig. 5). In the double *iscR ryhB* mutant, P*_erpA_*-*lacZ* expression levels were at their maximum throughout all DIP concentrations tested.

**FIG 6:**
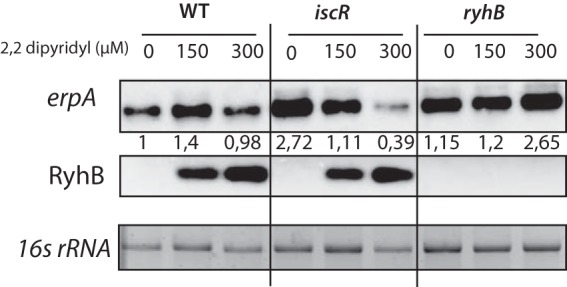
Expression of the *erpA* mRNA peaks at intermediate iron concentration. The MG1655 WT strain and its *ryhB* and *iscR* mutant derivatives were grown in LB containing either 0, 150, or 300 µM DIP to an OD_600_ of 0.6 before samples were prepared. Total RNA was extracted and submitted to a Northern blotting experiment with probes directed against the *erpA* mRNA, RyhB. The numbers below the top panel represent the relative fold changes compared to the 0-h time point in the WT strain. Quantification was made using the 16S RNA as a loading control. The gel depicted here is representative of 3 independent experiments.

To confirm these results, we performed Northern blotting experiments on the *erpA* mRNA by growing the cells either in the absence of DIP or with 150 µM or 300 µM DIP (Fig. 6). In a manner parallel to the P*_erpA_*-*lacZ* fusions described above, expression of *erpA* peaked in the presence of 150 µM DIP (1.5-fold compared to that in the absence of DIP) and remained minimal both in untreated cells and in cells treated with 300 µM DIP. In the *ryhB* mutant, *erpA* mRNA levels were similar to that of the WT strain at DIP concentrations of ≤150 µM but increased significantly at 300 µM DIP (2.5-fold higher than in untreated cells). In the *iscR* mutant strain, expression was maximal without DIP (2.5-fold higher than in the WT strain under the same conditions) and then decreased at 150 µM and 300 µM DIP. Of note, the regulatory effects seen on *erpA* mRNA levels are greater in Northern blotting experiments than with the translational fusion. This suggests that additional elements in the mRNA not present in the *lacZ* fusion may determine its degradation rate. An alternative explanation is that, in the β-galactosidase assays, the time after addition of DIP was too short to allow the preexisting pool of β-galactosidase to be diluted out by cell growth, masking the true extent of the effects.

Altogether, these results showed that the double IscR/RyhB coregulation allows maximal expression of *erpA* at concentrations of DIP ranging from 100 to 200 µM, with a peak at 150 µM DIP. At DIP concentrations lower than 100 µM, IscR represses *erpA* transcription, while at DIP concentrations above 200 µM, *erpA* expression is repressed by RyhB.

### Coregulation by IscR and RyhB is linked to ATC functional redundancy.

Previous work from our laboratory has shown that depending upon the growth conditions, defects in one ATC can be compensated by expression of another one (11). We thus hypothesized that the double RyhB/IscR regulation may serve to repress expression of *erpA* when other ATCs are present to sustain Fe-S delivery. Previous genetic analysis has revealed that SufA is used under iron-limiting conditions (21). Therefore, we predicted that allowing *erpA* expression under these conditions would allow cells to grow even in the absence of *sufA.*

To test this, we used *erpA* alleles containing the previously described *cis* mutations that hampered either IscR or RyhB regulation, i.e., *erpA*(RyhB^ind^) and *erpA*(IscR^ind^). After overnight growth in LB, cells were inoculated in fresh medium containing increasing concentrations of DIP (from 0 to 300 µM), and growth was followed for 14 h. We first did a control experiment by testing growth of strains carrying the *erpA*(RyhB^ind^) or the *erpA*(IscR^ind^) allele. Introducing either of the *erpA* alleles had no significant effect on growth of the WT strain (see Fig. S1 in the supplemental material).

**FIG 7:**
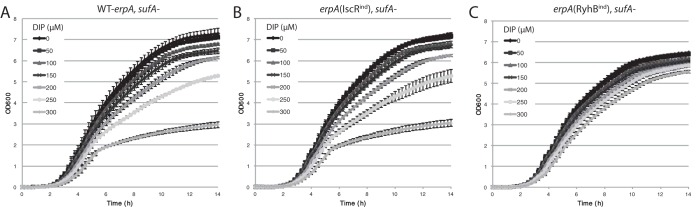
Growth of a *sufA* mutant in DIP is rescued by alleviating RyhB repression on *erpA*. The *sufA* mutant strains in which *erpA* is regulated by both RyhB and IscR (A) or for which repression by IscR (B) or RyhB (C) has been alleviated were tested for their growth in LB containing various concentrations of DIP. Overnight cultures of the different strains were diluted (1/1,000) in 100 µl LB containing increasing concentrations of DIP (0 to 300 µM) in microtiter plates, and growth was followed for 14 h in a microtiter plate reader. Error bars represent the standard deviations calculated for a minimum of four experiments.

Introducing the *sufA* mutation in either the *erpA*^+^ or *erpA*(IscR^ind^) strain severely affected growth when DIP was present at concentrations higher than 150 µM (Fig. 7A and B). Strikingly, introducing the *erpA*(RyhB^ind^) allele in the *sufA* mutant almost completely suppressed that growth defect (Fig. 7C). This result showed that alleviating repression of *erpA* compensates for the lack of SufA during iron starvation and that RyhB-mediated regulation prevents ATC redundancy.

## DISCUSSION

In the present study, we show that both the sRNA RyhB and the transcriptional regulator IscR repress expression of *erpA*, encoding the ATC transporter of Fe-S clusters essential under aerobic conditions. Interestingly we show that RyhB and IscR act under opposite conditions in regard to Fe concentration: IscR represses *erpA* when iron is abundant (a condition under which RyhB is not expressed), while RyhB represses *erpA* when iron is low (a condition under which IscR repression is alleviated). Under severely iron-depleted conditions (i.e., at high DIP concentrations), posttranscriptional repression by RyhB is compensated for by an increased transcription of the promoter that is no longer bound by holo-IscR. This phenomenon may provide an explanation of why regulation of *erpA* by RyhB was not found in previous, even recent, global transcriptomic approaches (20, 24). We propose that this mechanism enables expression of *erpA* when it is most needed and turns it down when other, functionally redundant ATCs are able to sustain Fe-S protein maturation.

**FIG 8:**
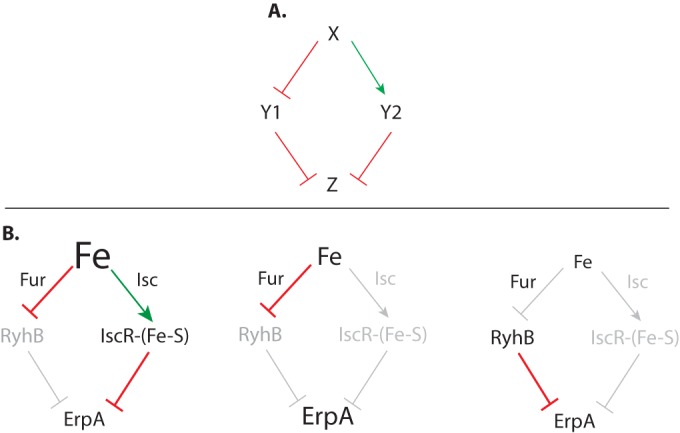
Incoherent regulation prevents ATC redundancy. (A) Schematics of an incoherent diamond circuit: signal X represses Y1 and activates Y2; both Y1 and Y2 are repressors of Z. (B) A simplified model for the regulation of *erpA* showing its resemblance to the incoherent diamond depicted in panel A. (Left) At high [Fe], Fur represses RyhB and Isc stimulates the formation of IscR-[Fe-S], which represses expression of *erpA*. (Middle) At intermediate [Fe], neither RyhB nor IscR-[Fe-S] is produced and expression of *erpA* is maximal. (Right) At low [Fe], IscR is in its apo form, thereby alleviating its repression of *erpA* at the transcriptional level, but RyhB is expressed and represses *erpA* expression.

The regulatory mechanism that we unraveled here for expression of *erpA* is best described as an “incoherent” circuit (30, 31). In such a circuit, a common signal generates two antagonistic effects (i.e., activation and repression) on the expression of a target gene (Fig. 8A) (32). Here, Fe concentration acts as a common signal perceived by both IscR and RyhB, with two antagonistic outcomes on the expression of *erpA*: Fe deprivation yields to RyhB repression and to induction by alleviation of holo-IscR repression (Fig. 8B).

Incoherent circuit motifs have been shown to drive nonmonotonic responses, also called biphasic responses, to an inducer (28). Consistently, *erpA* levels are not linearly correlated with Fe levels but instead peak within a narrow iron concentration range (Fig. 5 and 6). To our knowledge, only one other naturally occurring incoherent circuit giving rise to a biphasic response has been documented in *E. coli*: that of the *galKETK* operon, which is dually regulated by cyclic AMP (cAMP) receptor protein (CRP) and GalS (29). In this case, *gal* expression peaks at intermediate levels of the signal cAMP, but the physiological advantage of this peak remains elusive.

The biphasic regulation of *erpA* expression can be appreciated within the context of Fe-S cluster homeostasis throughout iron concentration fluctuation, as this occurs in natural settings. *E. coli* has two systems, ISC and SUF and a series of ATCs, which make and deliver Fe-S clusters to target proteins, respectively. Over 100 Fe-S cluster-containing proteins are predicted to arise in *E. coli*, making Fe-S cluster-based processes high Fe consumers. Previous work showed that ISC does not function at low iron concentrations while SUF is not synthesized at high iron concentrations (7). For instance, our work and that of Outten et al. (21) show that *suf* mutants are impaired in growth at DIP concentrations of ≥200 µM, strongly suggesting that this constitutes a turning point in the ISC-to-SUF utilization (Fig. 7C and reference 21). Strikingly, *erpA* expression is maximized precisely at those same DIP concentrations (150 µM to 200 µM) where cells shift from ISC to SUF, as a result of the regulation that we here deciphered (Fig. 5). Thus, biphasic expression of *erpA* explains how *E. coli* coordinates ATC usage. Indeed, a *cis* mutation abrogating RyhB-mediated inhibition of ErpA was sufficient to compensate for the absence of SufA under severe iron limitation. This demonstrates that, under such iron limitation, both SufA and ErpA can be equally efficient ATCs but that the cell ought to use only one to optimize utilization of the scarce iron available. To do so, the cell relies on the IscR/RyhB dual control here described.

We note that neither IscR nor RyhB repression of *erpA* expression is total and that, at any given iron concentration, production of *erpA* is maintained (Fig. 5). This is most likely because ErpA is essential under aerobiosis conditions to sustain Fe-S delivery to its essential targets IspG/H. Interestingly, a recent ribosome profiling study has shown that under iron-sufficient conditions ErpA is present at relatively high levels in the cell (33). We propose that this regulatory circuit results from an evolutionary tradeoff between the essentiality of ErpA and the need to limit its expression under conditions for which other ATCs are at least partially redundant for target maturation. ErpA being present at a high level in the cell, it is likewise a high Fe user. Therefore, it seems reasonable to propose that one of its negative regulators, presumably RyhB, appeared first during evolution to avoid unnecessary Fe utilization. However, as ErpA became essential under aerobiosis, a second opposing regulator, IscR, was incorporated to counter the effect of the first, thus allowing the two constraints to be met: sparing iron at the cellular level and filling in the ErpA essential function.

Remarkably, the regulatory circuit behind *erpA* expression is a mixed regulatory circuit, comprising a transcriptional regulator and an sRNA. The mixed composition provides *E. coli* with a series of advantages. First, the recruitment of the sRNA RyhB is critical in providing “incoherence” to the circuit. Indeed, repression under low-iron conditions could not have been achieved by Fur and IscR, as they are active under high iron levels. The use of an sRNA (here RyhB) as an intermediate allows negative repression to arise at a low iron level as well. Second, use of both protein- and RNA-mediated regulation allows separation of their activities more effectively without much chance of cross-interference between the two. This was best shown here by the possibility of suppressing IscR regulation of *erpA* without affecting RyhB regulation (and vice versa). Uncoupling the regulatory levels very likely provides more flexibility on the tuning of the response, as affinity for one binding site of the target gene can be changed independently of the other. Third, using a mixed circuit may have effects on the dynamics of the response. For instance, RNA regulation is assumed to be faster than transcriptional regulation (34). Future studies will aim at studying the dynamic response of *erpA* expression when switching from high- to low-iron conditions.

The complex relationship between transcription factors and RNA regulators is an area of research that is in expansion (see references 26 and 35 for reviews). Examples of such relationships include sRNAs that are regulated by well-known two-component systems and that regulate these systems in return (e.g., OmrA/B and EnvZ-OmpR [36]) or major regulators that participate in so-called mixed circuit motifs with sRNAs (e.g., Spot42/CRP [37]). However, how mixed circuits differ from circuits composed only of transcriptional regulators is still only partially understood. We believe that our study helps shed some light on the possible advantages of mixed regulatory circuits.

## MATERIALS AND METHODS

### Strains and culture.

All strains used in this study are derivative of *E. coli* MG1655 and are listed in Table S1 in the supplemental material. Strains were grown in LB broth (Sigma), containing various concentrations of 2,2′-dipyridyl (DIP) (Sigma) when stated. Marked mutations were moved between strains using classical P1 phage transduction as previously described (38). The plasmid library and the plac and pRyhB plasmids used in this study are described in reference 25. Transformations were carried out as described previously (39). PCR amplifications were carried out using the GoTaq DNA polymerase from Promega.

For the growth assay presented in Fig. 7, overnight cultures of the different strains were diluted (1/1,000) in individual wells of a 96-well microtiter plate in 100 µl LB containing increasing concentrations of DIP. Cells were then grown at 37°C, with agitation, in a Tecan Infinite 200 microtiter plate reader. OD_600_ was measured every 15 min without removing the plate from the machine, and growth was followed for 14 h.

### Genetic manipulations.

The P_BAD_-*erpA-lacZ* and P*_erpA_-lacZ* fusions were constructed using recombination in a specifically designed strain, as previously described (39). Briefly, sequences corresponding to the *erpA* gene starting from its +1 transcriptional start site or from −200 nt before the +1 site up to 30 nt downstream of the ATG codon were amplified using oligonucleotides P_BAD_-erpA-F and LacZ-erpA-R or Perpa(-200)-F and LacZ-erpA-R, respectively. The purified PCR products were then electroporated into strain PM1205 for recombination. Recombinants carrying the desired fusion were selected on LB plates devoid of NaCl and containing 5% sucrose and 40 µg/ml X-Gal (5-bromo-4-chloro-3-indolyl-β-d-galactopyranoside). Blue colonies were chosen, and the resulting fusions were sequenced using oligonucleotides lacI-F and Deep-lac.

Constructions of point mutations of the P_BAD_-*erpA-lacZ* fusion or the P*_erpA_-lacZ* fusion were realized by an overlap PCR. For each mutant, two PCR products corresponding to the sequence upstream and downstream of the desired mutation were amplified by PCR with oligonucleotides containing the desired mutation and using the strains containing the WT fusions as the templates. The two PCR products were then joined by an overlap PCR using oligonucleotides lacI-F and Deep-lac. The resulting PCR products were purified and electroporated in strain PM1205 as described above.

The chromosomal point mutations on *erpA* designed to abrogate regulation by IscR or RyhB were constructed as follows. First, PCR products containing the desired point mutations were realized using overlap PCR with oligonucleotides yadQ-F and erpA-mut-1R and erpA-mut1-F and erpA-R [for the *erpA*(RyhB^ind^) mutation] and oligonucleotides yadQ-F and erpA-iscR-R and erpA-iscR-F and erpA-R [for the *erpA*(IscR^ind^) mutation], using MG1655 as a template. The PCR products were then electroporated in strain LL401, in which the *erpA* promoter had been previously replaced by a P_BAD_ promoter. The LL401 strain had previously been transformed with mini-Lambda Red in order to allow recombination with PCR products. Recombinants were selected by plating the cells on LB plates containing 0.2% glucose. As *erpA* is essential, strain LL401 is unable to grow in the presence of glucose. Only clones that had lost the P_BAD_ promoter by recombining with the PCR products were thus selected. The obtained clones were then sequenced using oligonucleotides yadQ-F and erpA-R.

Mutations in the pRyhB plasmid were obtained as follows. In a first step, the pRyhB plasmid, purified from a WT *E. coli* strain, was amplified by PCR with oligonucleotides RyhB1.2-F and RyhB1.2-R, containing the desired mutation. The resulting PCR product was digested with the DpnI enzyme for 1 h at 37°C to get rid of the native plasmid.

### RNA extraction and Northern blotting experiments.

Overnight cultures of the appropriate strains were diluted in fresh medium containing ampicillin when indicated and incubated at 37°C with agitation. At an OD_600_ of 0.5, 1-ml samples of the cultures were extracted and 2,2′-dipyridyl and/or IPTG was immediately added to the culture before new samples were extracted at indicated time points. RNA was extracted from the samples using the hot-phenol method as previously described (27) and resuspended in 10 µl diethyl pyrocarbonate (DEPC)-treated water final. Total RNAs were run on 1.75% agarose denaturing gels. RNA was then transferred onto Zeta Probe (Bio-Rad) positively charged membranes by an overnight reversed capillary transfer. Transferred RNAs were cross-linked to the membrane using a UV cross-linker. Membranes were hybridized with specific biotinylated probes overnight at 42°C, and RNAs were detected using the North2South (Thermo Scientific) labeling kit according to the manufacturer’s instructions. Biotinylated oligonucleotide probes against the *erpA* mRNA, SsrA, and RyhB were ordered from Eurofins (see Table S2 in the supplemental material).

### β-Galactosidase assays.

For microtiter plate assays, overnight cultures of the specified strains were diluted 500-fold into 100 µl of fresh medium (containing ampicillin and IPTG or DIP when indicated) contained in a well of a microtiter plate. The microtiter plates were then incubated at 37°C with agitation. β-Galactosidase assays were performed as previously described (26) with slight modification. After 6 h of growth, the OD_600_ of the cultures were read using a Tecan Infinite 200 reader (Tecan). Cells were lysed by adding 50 µl of permeabilization buffer and incubated for 15 min at room temperature.

*o*-Nitrophenyl-β-d-galactopyranoside (ONPG) was then added to the lysed cells, and degradation of ONPG by the β-galactosidase was followed by measuring the OD_420_ in a kinetic manner on a Tecan Infinite 200 reader. Specific activities were calculated by dividing the *V*_max_ of the appearance of the OD_420_ by the OD_600_. Values obtained were multiplied by 100,000 to give values that approximate Miller units (empirically determined).

## SUPPLEMENTAL MATERIAL

Figure S1  Strains in which *erpA* is regulated by both RyhB and IscR (A) or for which repression by IscR (B) or RyhB (C) has been alleviated were tested for their growth in LB containing various concentrations of DIP. Overnight cultures of the different strains were diluted (1/1,000) in 100 µl LB containing increasing concentration of DIP (50 to 300 µM) in microtiter plates, and growth was followed for 14 h in a microtiter plate reader. Error bars represent the standard deviations calculated for a minimum of four experiments. Download Figure S1, PDF file, 0.6 MB

Table S1  Strains used in this study Table S1, DOCX file, 0.1 MB

Table S2  Oligonucleotides used in this study Table S2, DOCX file, 0.1 MB
