# Supplementary material for: A Regulatory Circuit Composed of a Transcription Factor, IscR, and a Regulatory RNA, RyhB, Controls Fe-S Cluster Delivery
Source: mBio. 2016 Sep 20;7(5):e00966-16. doi: 10.1128/mBio.00966-16 (PMC5040110; doi:10.1128/mBio.00966-16)
Supplement: Figure S1 — Strains in which erpA is regulated by both RyhB and IscR (A) or for which repression by IscR (B) or RyhB (C) has been alleviated were tested for their growth in LB containing various concentrations of DIP. Overnight cultures of the different strains were diluted (1/1,000) in 100 µl LB containing increasing concentration of DIP (50 to 300 µM) in microtiter plates, and growth was followed for 14 h in a microtiter plate reader. Error bars represent the standard deviations calculated for a minimum of four experiments. Download [file mbo004162985sf1.pdf]

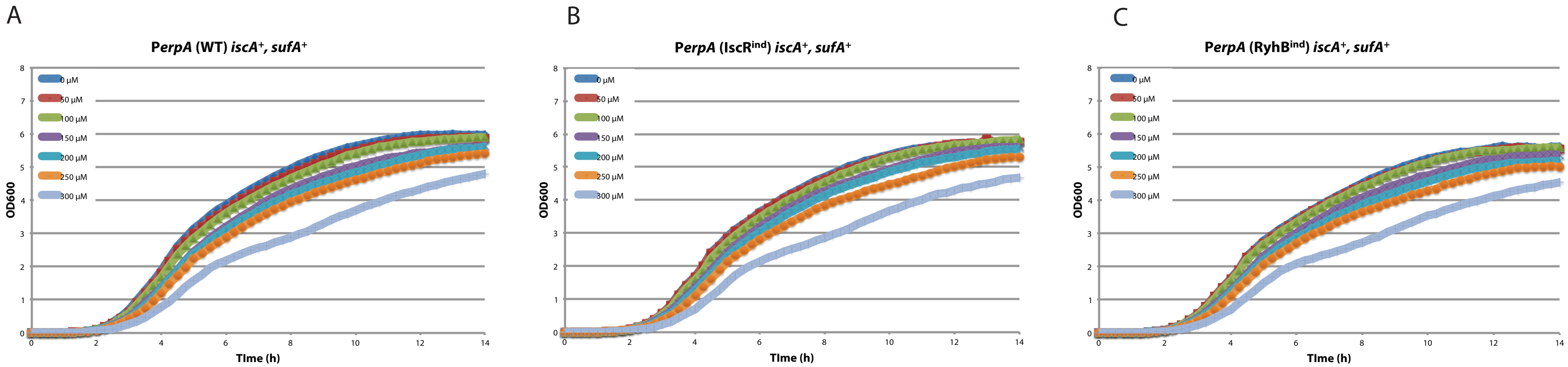
A
PerpA (WT) iscA+, sufA+
B
PerpA (IscRind) iscA+, sufA+
C
PerpA (RyhBind) iscA+, sufA+
0 µM
50 µM
100 µM
150 µM
200 µM
250 µM
300 µM
OD600
TIme (h)
