# Supplementary material for: A Regulatory Circuit Composed of a Transcription Factor, IscR, and a Regulatory RNA, RyhB, Controls Fe-S Cluster Delivery
Source: mBio. 2016 Sep 20;7(5):e00966-16. doi: 10.1128/mBio.00966-16 (PMC5040110; doi:10.1128/mBio.00966-16)
Supplement: Table S1 — Strains used in this study [file mbo004162985st1.docx]

**Table S1 : Strains used in this study**

| Strain Name | Description | Reference |
| --- | --- | --- |
| MG1655 | *E. coli* WT | Laboratory strain |
| PM1205 | MG1655 *mal*::*lacI^q^, ΔaraBAD, lacI'*:: P*_BAD_-cat-sacB:lacZ, mini λ tet^R^* | [1] |
| PM1470 | PM1205 *lacI'::*P*_BAD_-erpA-lacZ* | This study |
| PM2010 | PM1470 *ryhB ::cat* | This study |
| PM2013 | PM1470 *iscR ::kan* | This study |
| PM2014 | PM1470 *ryhB ::cat, iscR ::kan* | This study |
| PM2030 | PM1205 *lacI'::*P*_BAD_-erpA(mut1)-lacZ* | This study |
| PM2031 | PM1205 *lacI'::*P*_BAD_-erpA(mut2)-lacZ* | This study |
| PM2034 | PM1205 *lacI'::*P*_BAD_-erpA(mut3)-lacZ* | This study |
| PM2035 | PM1205 *lacI'::*P*_BAD_-erpA(mut4)-lacZ* | This study |
| PM2036 | PM1205 *lacI'::*P*_BAD_-erpA(mut5)-lacZ* | This study |
| PM2040 | PM1205 *lacI'::*P*_erpA_-erpA-lacZ* | This study |
| PM2041 | PM2040 *ryhB ::cat* | This study |
| PM2042 | PM2040 *iscR ::kan* | This study |
| PM2043 | PM2040 *ryhB ::cat, iscR ::kan* | This study |
| PM2044 | PM1205 *lacI'::*P*_erpA_-erpA*(IscR^ind^)*-lacZ* | This study |
| PM3060 | PM2044 *ryhB ::cat* | This study |
| PM3061 | PM2044 *iscR ::kan* | This study |
| PM3062 | PM2044 *ryhB ::cat, iscR ::kan* | This study |
| PM2046 | PM1205 *lacI'::*P*_erpA_-erpA*(RyhB^ind^)*-lacZ* | This study |
| PM3063 | PM2046 *ryhB ::cat* | This study |
| PM3064 | PM2046 *iscR ::kan* | This study |
| PM3065 | PM2046 *ryhB ::cat, iscR ::kan* | This study |
| PM3001 | MG1655 *ryhB ::cat* | This study |
| SC115 | MG1655 *iscR ::kan* | This study |
| SC107 | MG1655 *iscR ::kan, ryhB ::cat* | This study |
| LL401 | *∆PerpA :: spc*  P*_BAD_ ::erpA* | [2] |
| PM3050 | LL401 P*erpA*(WT)-*erpA* | This study |
| SC063 | PM3050 *iscA ::cat* | This study |
| SC067 | PM3050 *sufA ::kan* | This study |
| PM3051 | LL401 P*erpA*(IscR^ind^)-*erpA* | This study |
| SC064 | PM3051 *iscA ::cat* | This study |
| SC068 | PM3051 *sufA ::kan* | This study |
| PM3053 | LL401 P*erpA*(RyhB^ind^)-*erpA* | This study |
| SC065 | PM3053 *iscA ::cat* | This study |
| SC069 | PM3053 *sufA ::kan* | This study |

1. Mandin P, Gottesman S. A genetic approach for finding small RNAs regulators of genes of interest identifies RybC as regulating the DpiA/DpiB two-component system. Mol Microbiol. 2009;72: 551–565.

2. Loiseau L, Gerez C, Bekker M, Ollagnier-de Choudens S, Py B, Sanakis Y, et al. ErpA, an iron sulfur (Fe S) protein of the A-type essential for respiratory metabolism in Escherichia coli. Proc Natl Acad Sci U S A. 2007;104: 13626–13631.
