# Supplementary material for: A Regulatory Circuit Composed of a Transcription Factor, IscR, and a Regulatory RNA, RyhB, Controls Fe-S Cluster Delivery
Source: mBio. 2016 Sep 20;7(5):e00966-16. doi: 10.1128/mBio.00966-16 (PMC5040110; doi:10.1128/mBio.00966-16)
Supplement: Table S2 — Oligonucleotides used in this study [file mbo004162985st2.docx]

**Table S2 : Oligonucleotides used in this study**:

| Name | 5’ to 3’ sequence |
| --- | --- |
| PBAD-erpA-F | ACCTGACGCTTTTTATCGCAACTCTCTACTGTTTCTCCATATTATTGGGTTAGAATTTGC |
| lacZ-erpA-R | taacgccagggttttcccagtcacgacgttgtaaaacgacCTCCAGCGGCAGTGCTACGT |
| Perpa(-200)-f | CGAAGCGGCATGCATTTACGTTGACACCATCGAATGGCGCTAGCGCAATTTACCGGCG |
| deeplac | CCGGGCCTCTTCGCTA |
| lacIF | TACGTTGACACCATCGAATGG |
| erpa-mut1-F | GATGTCGTACCGAGCAAAATatgAGTGATGAC |
| erpa-mut1-R | ATTTTGCTCGGTACGACATCGGCAATTGGGCA |
| erpa-mut2-F | CGTTTGGACGAAAATatgAGTGATGACGTAG |
| erpa-mut2-R | catATTTTCGTCCAAACGACATCGGC |
| erpa-mut3-F | AAAATatgTCAGATGACGTAGCACTGCCGCTG |
| erpa-mut3-R | ACGTCATCTGAcatATTTTGCTCCAAACGAC |
| erpa-mut4-F | AGTGATGAGCTAGCACTGCCGCTGGAG |
| erpa-mut4-R | CAGTGCTAGCTCATCACTcatATTTTGCTC |
| erpa-mut5-F | TAGCACTGGGCCTGGAGGTCGTTTTACAAC |
| erpa-mut5-R | ACCTCCAGGCCCAGTGCTACGTCATCACTc |
| yadQ-F | TAGCGCAATTTACCGGCGGG |
| erpA-R | TCATATCGCCTTCGTTCACC |
| erpA-iscR-F | ATAGTAGCTGCAGTAGATAATGGCGATTATTATTGGG |
| erpA-iscR-R | CTACTGCAGCTACTATTTCGTtcaAGTATTCTC |
| RyhBI.2F | agcacgacattgctcacattcgttccagtattacttagcc |
| RyhBI.2R | ggctaagtaatactggaacgaatgtgagcaatgtcgtgct |
| RyhB-NB | AAGTAATACTGGAAGCAATGTGAGCAATGTCGTGCTTTCAGGTTCTC |
| SsrA-NB | CGCCACTAACAAACTAGCCTGATTAAGTTTTAACGCTTCA |
| ErpA-NB | TTTTAACTTTGTTGGCTGCTGCGTCGGTAAACTCCAGCGGCAGTGCTACG |
